# Gun-Shot Wound of the Axillary Artery—Diffused Aneurism—Ligature of Subclavian Artery—Death on Ninth Day from Pleuritis

**Published:** 1864-09

**Authors:** Wm. Selden

**Affiliations:** Surgeon Liberty Hospital.


ART. V.—*Gun-Shot Wound of the Axillary Artery—Diffused Aneurism—Ligature of Subclavian Artery—Death on ninth day from Pleuritis.* By WM. SELDEN, Surgeon Liberty Hospital.

Private K. P. Kahea, company "B," Jeff. Davis Legion, aged 29—very large and muscular—while acting as scout, near the Peaks of Otter, on June 14th, was shot with a minie ball through the left axilla; hemorrhage represented as very profuse, notwithstanding which he rode eight miles, closely pursued for three miles. He spent several days in a private house, and was admitted to Campbell's Hospital on 19th. The ball had passed through the tendons of the pect. maj. and lat. dorsi, severing the axillary artery, apparently in its lower third; the hemorrhage had ceased spontaneously on the first day and had not recurred; pulse imperceptible; very great swelling and hardness in the axilla, extending to the elbow, with great discoloration from ecchymosis; severe pain from shoulder to hand, with a sense of numbness, but not complete loss of sensation; the capillary circulation but little impaired, and temperature normal; when he sat up the veins of the forearm became much distended; wounds healthy and healing; pulse in the right arm feeble and frequent—above 100; appetite feeble; he slept but little, and then from the influence of opium. He continued in the same state, with little variation, for three weeks; sometimes we thought that we could feel a faint pulsation in the radial artery, but it was so slight as to be doubtful. Early in July, while the general swelling of the arm diminished, the tumor in the axilla was obviously enlarging and extending under the pectoral muscle, when, by the 8th, it became very prominent and as large as the fist. On the night of the 10th, a free arterial hemorrhage took place from the posterior wound; after the loss of about a pint of blood, it was arrested by pressure for an hour upon the subclavian above the clavicle, and did not return; his pulse very feeble, and above 120; he was very much alarmed about his condition—indeed, he had been unusually low spirited from the first. On the 11th, for the first time, a distinct pulsation was felt in the tumor, both in the axilla and over the pectoral muscle; there was no perceptible thrill or bruit; from this time the tumor steadily increased in size, and the pulsation daily became stronger; there was also increase of the pain and numbness in the limb; constant fever and sleeplessness and loss of appetite. It was decided, in consultation, to tie the subclavian above the clavicle, as affording him the best chance for recovery, although his general condition was not favorable for an operation. Accordingly, on the 23d July, assisted by Surgeon Blackford and the rest of the surgical staff of this post, I ligated the artery where it passes over the first rib. The operation was rendered somewhat difficult by the unusual number of superficial arteries that required to be tied, and by the elevation of the clavicle from the tumor in the axilla. The pulsation in the tumor immediately ceased, and did not return; the swelling became less tense, but the pain continued and the fever increased; the capillary circulation in the limb continued good, and its temperature appeared to be little if at all diminished, (we had no thermometer to test it accurately,) for a few days he seemed doing pretty well, but on the 26th, the incision presented an unhealthy appearance, with a slight erysipelatous blush and some swelling below the clavicle. By the 28th, the shoulder and breast became enormously swollen, so as completely to conceal the aneurismal tumor. On the next day, 29th, there was extensive erysipelas on the outside and back of shoulder, which spread rapidly over the breast and down the arm to the elbow; the incision suppurating and unhealthy. On the same day, he was seized with a severe pleuritic pain on left side, and great difficulty of breathing, but without cough; the respiratory motion was confined so exclusively to the right side that the left seemed paralyzed, and was obviously several inches smaller than the right side, although auscultation showed the presence of effusion in the left thorax; bowels torpid and tympanitic; pulse 150 and very feeble. 30th. No improvement in his condition, although the pain in the side had nearly ceased. 31st. Prostration extreme; respiration more difficult; died soon after midnight.

*Autopsy.*—Axillary artery and vein both severed by the ball in their lower third; the axilla filled with a large clot extending to within three inches of the elbow and considerably beneath the pector. maj. The coagulum was moderately firm and contained in a thin adventitious sac of cellular tissue, but without any fibrinous deposite. The median nerve had escaped division, but was very much discolored, as were also the other nerves in the axilla. The artery, where ligated, had united, but not very firmly; no clot had formed within it, owing probably to the fact that the posterior scapular artery, instead of being a branch from the transversalis colli, arose directly from the subclavian, between the scaleni, and about two-thirds of an inch above the point of ligation; this would probably have led to secondary hemorrhage after the separation of the ligature. There was a large serous effusion in the left side of the thorax, with a deposite of a thick layer of fibrine over a large surface of the lung; phrenic nerve healthy. There was also slight deposit in the pericardium and some effusion. The only treatment that was admissable after the operation was morphine, stimulants and tinct. mur. ferri.

It would probably have been better to have tied the subclavian soon after his admission, when his general health was less impaired. But would the rules of surgery have justified the ligation of a large artery when there was no hemorrhage and no pulsation in the tumor? The axillary swelling and absence of pulse at the wrist afforded strong presumptive evidence that the artery was divided, but we could not be sure that the absence of pulse was not owing to the pressure of the tumor, which might have arisen from the division of a branch of the axillary, and if so we might reasonably hope that in time it would be absorbed and the circulation restored. It was not until the tumor began to increase in size, with distinct pulsation, that we felt satisfied that an operation was indispensable, and our choice then lay between disarticulation and ligature of the subclavian—the ligature of the axillary in the midst of such swelling and altered relation of the parts was out of the question. We decided upon the ligation of the artery as being sanctioned by the highest authority; the more especially as his constitutional condition almost forbade the hope of successful amputation.

I would suggest, that in a similar case, where the posterior scapular arose directly from the subclavian, it would be proper to tie it, as well as the main artery; in this case it could have been done without difficulty, as it could be plainly seen and felt where it crossed the cervical plexus. The other branches of the transverse colli and the supra-scapular would probably be sufficient to supply the anastomosing circulation. The immediate cause of death in this case was pleuritis, which has been observed to be far the most frequent cause of death after ligation of subclavian. The erysipelas to which there has latterly been some tendency in this neighborhood, no doubt also contributed to the fatal termination.

